# Feasibility and Safety of Office-Based Transnasal Balloon Dilation for Neopharyngeal and Proximal Esophageal Strictures in Patients with a History of Head and Neck Carcinoma

**DOI:** 10.1007/s00455-021-10253-z

**Published:** 2021-03-10

**Authors:** Anouk S. Schimberg, David J. Wellenstein, Henrieke W. Schutte, J. Honings, Henri A. M. Marres, Robert P. Takes, Guido B. van den Broek

**Affiliations:** Department of Otorhinolaryngology and Head and Neck Surgery, Radboud University Medical Center, Postbus 9101, 6500 HB, Nijmegen, The Netherlands

**Keywords:** Office-based, Transnasal balloon dilatation, Topical anesthesia, Esophageal strictures, Head and neck carcinoma

## Abstract

The aim of this study was to assess feasibility and safety of office-based transnasal balloon dilation of neopharyngeal and proximal esophageal strictures in patients with a history of head and neck carcinoma. The secondary objective was to explore its effectiveness. This prospective case series included patients previously treated for head and neck carcinoma with neopharyngeal or proximal esophageal strictures who underwent transnasal balloon dilation under topical anesthesia. The target dilation diameter was 15 mm; if necessary dilation procedures were repeated every 2–4 weeks until this target was reached. Completion rates, adverse events, and patient experiences measured by VAS scores (0 = no complaints – 10 = unbearable complaints), dysphagia scores based on food consistency (0 = no dysphagia – 5 = unable to swallow liquids/saliva), and self-reported changes in swallowing symptoms were recorded. Follow-up was 2 months. Twenty-six procedures were performed in 12 patients, with a completion rate of 92%. One minor complication occurred, i.e. an infection of the dilation site. Tolerance of the procedure was good (median VAS = 2). The dysphagia score improved after a mean of 2.2 procedures per patient, however not significantly. Eight patients reported improvement in dysphagia, of whom 3 had recurrence of dysphagia within 1 month post-treatment. Office-based transnasal balloon dilation is a feasible and safe in-office procedure which is well-tolerated by patients. The dilations can improve dysphagia, although effects might be transient.

## Introduction

After the introduction of ultrathin transnasal esophagoscopes, office-based transnasal balloon dilation under topical anesthesia emerged as a novel treatment for pharyngeal and esophageal strictures. These strictures are a common cause of dysphagia in patients previously treated for head and neck carcinoma. Stricture formation occurs in up to 19% of laryngectomy patients and in 17% of patients who have been treated with intensity-modulated radiotherapy on the head and neck area. However, these numbers are based on patients treated between 1989 and 2009, and therefore may be outdated [1, 2].

Traditional methods for pharyngeal and esophageal dilation are transoral balloon dilation and bougienage, either under general anesthesia (GA), under sedation or in the awake patient [3]. Bougienage can also be performed as self-dilation by the patient [4, 5]. A recent meta-analysis of Josino et al. described similar success and complication rates for both methods and less postprocedural pain after balloon dilation of benign esophageal strictures [6]. The disadvantage of the transoral techniques is that, because of the gag reflex, not all patients tolerate the procedure. For self-dilation also holds that it can only be performed in a selected group of patients, as the patient should be able to understand and perform the procedure, and not all strictures are suitable for self-dilation, depending on its morphology [4, 5, 7, 8].

By transitioning the approach from transoral to transnasal using a thin flexible endoscope with working channel, gag reflex is less prominent or even absent. Tolerability of transnasal esophagoscopy (TNE) is good [9]. Transnasal balloon dilation can be performed as an extension of TNE and thereby facilitates the performance of dilation under topical anesthesia in the office. The omittance of general anesthesia decreases health risks for patients and hospital costs [10]. This is important because two thirds of the patients require repeated interventions due to transient treatment effects [11]. Another advantage is that patients with anatomic limitations hindering treatment with bougienage using rigid endoscopy under GA, such as severe trismus or a limited neck extension due to previous oncologic therapy, have an alternative. Performing dilations in awake patients results in direct feedback of the patient, and may reduce postprocedural pain and complication risks.

Office-based transnasal balloon dilation of neopharyngeal and proximal esophageal strictures was first described by Rees in 2007[12] and evaluated in two retrospective clinical reports [13, 14]. Howell et al. [14] exclusively included patients with a history of head and neck carcinoma, while Rees et al. [13] also included patients with other benign strictures. Both studies demonstrated that the procedure was well-tolerated by patients and safe, as no major complications occurred. Recently, a systematic review on dilation procedures in head and neck cancer patients revealed a success rate of 72.9% (using heterogeneous definitions of success), repeated intervention rate of 63%, and a complication rate of 4.4%, concerning perforations in 50% of the complications [11]. Because this review concerned exclusively bougie and balloon dilations performed under GA, these data cannot be used for balloon dilations performed in the awake patient.

In this first prospective study on office-based transnasal balloon dilation of neopharyngeal and proximal esophageal strictures in patients with a history of head and neck carcinoma, we hypothesize that this procedure is feasible and safe. To address this primary objective, feasibility was measured by completion rate and patients’ subjective experiences, and safety by complication rate. Effectiveness of office-based transnasal balloon dilation under topical anesthesia has never been assessed in previous studies. Therefore, the secondary objective of this study was to explore the effectiveness by reporting changes in dysphagia score and changes in swallowing complaints reported by the patient before and after transnasal balloon dilation.

## Materials and Methods

### Study Design and Patient Selection

This prospective case series was conducted from May 2018 until September 2019 at the department of Otorhinolaryngology and Head and Neck Surgery in a tertiary referral head and neck center in The Netherlands. Eligible study participants were adult patients with a history of head and neck carcinoma having symptoms of dysphagia caused by a neopharyngeal or proximal esophageal stricture. Additionally, patients had to have a sufficiently patent nasal cavity for the transnasal esophagoscope to pass through. Patients in whom general anesthesia was contra-indicated due to comorbidities and patients who preferred to undergo dilation under topical anesthesia were included. Written informed consent was acquired from each patient.

### Dilation Procedure

Transnasal balloon dilation was performed in the outpatient clinic under topical anesthesia. First, both nasal cavities were anesthetized with cotton pledgets soaked in 10% lidocaine spray and 0.1% xylometazoline solution. After 10–15 min, these pledgets were removed. Subsequently, a maximum of 10 sprays of 10% lidocaine was administered through the oropharynx to anesthetize the (laryngo-)pharyngeal region. A transnasal esophagoscope with 2 mm diameter working channel (EE1580K, Pentax Medical, Uithoorn, The Netherlands, diameter 5.1 mm) was introduced in the most patent nasal cavity and the stricture was visualized. Digital images were processed using a video processor (EPK-i5000-HD, Pentax Medical, Uithoorn, The Netherlands). If the stricture was large enough for the esophagoscope to pass, the entire esophagus was visualized. Four sizes of balloon dilators were available, i.e. 6–8, 8–10, 10–12, and 12–15 mm diameter, with a balloon length of 5.5 cm (CRE™ PRO Wireguided, Boston Scientific Corporation, USA). The balloon size was chosen based on the extent of the stricture, which can be estimated in reference to the endoscope tip (5.1 mm). Alongside the esophagoscope, the balloon dilator with guidewire was introduced in the nasal cavity and the balloon was positioned across the area to be dilated. By using a syringe filled with water placed in an integrated pressure gauge (Alliance™ II Integrated Inflation Device, Boston Scientific Corporation, USA), the balloon dilators were inflated under constant visualization while monitoring pressure and balloon size. After 1 min, the balloon was fully deflated. If the patient tolerated the first dilation, a second dilation was performed with 1 min between the inflations. At each subsequent inflation, the dilator diameter was increased with a maximum of 1.0–1.5 mm. In accordance with the clinical esophageal dilation guidelines, a maximum of 3 subsequent inflations in 1 session were performed [15]. After each inflation, the dilation site was inspected for mucosal injury. The procedure was aborted when mucosal defects occurred or when the patient did not tolerate further dilation because of pain or discomfort. The target diameter of the balloon was 15 mm because this luminal diameter is expected to be sufficient for patients to be relieved of dysphagia [15]. If this target was not reached after the dilation session and the patient was willing to undergo another dilation procedure, this was planned 2–4 weeks later. When a luminal patency of 15 mm was achieved, a follow-up consultation by phone was planned after 4 and 8 weeks. If the patient had recurring or persisting symptoms and was unwilling to undergo another transnasal dilation, alternative treatment options were discussed.

### Data Collection

Demographical data of the included patients were collected. At all pre- and post-procedural visits or consultations by phone, dysphagia grade was assessed using a score ranging 0–5: (0) normal swallowing function (no or minimal dietary modifications, normal duration of taking a meal, no aspiration) and no complaints of dysphagia; (1) normal swallowing function with intermittent complaints of dysphagia with solid food; (2) unable to swallow solid food; (3) unable to swallow minced food; (4) unable to swallow pureed / liquidized food; (5) unable to swallow clear liquids or saliva [16]. Data on the procedures were recorded, i.e. whether the endoscope could be passed through the stricture, the maximal diameter of dilations, the number of repeated dilation procedures, completion rate (if the patient had undergone 3 subsequent dilations in 1 session), and complications.

After each procedure, patients were requested to fill out a questionnaire concerning the procedural experiences. Patients were asked to rate their experiences according to pain in the nose and throat, complaints of gagging, ructus or nausea, and to give a general opinion on tolerability of the procedure. For this rating, VAS (visual analogue scale) was used with scores ranging from 0–10; 0 meaning no discomfort, 10 meaning unbearable discomfort.

For the data analyses, IBM SPSS statistics version 25 (IBM Corporation, Armonk, NY, USA) for Windows (Microsoft Corporation, Redmond, WA, USA) was used.

## Results

Twelve patients were included, in whom a total of 26 procedures were performed. The mean age was 72.2 years (SD 6.4). All patients had a history of head and neck carcinoma, 10 of whom previously underwent a total laryng(opharyng)ectomy (Table 1). More than half of the study population had a history of recurrent strictures of the neopharynx or proximal esophagus and previously underwent one or more dilations. Two procedures were discontinued after 1 or 2 (instead of 3) dilation inflations due to discomfort or pain. One patient developed an infection of the dilation site and was successfully treated with short term oral antibiotics and corticosteroids.

**Table 1:** Patient characteristics

Patient (sex, age)	Tumor site	Treatment	Previous dilations	Dilation site	Max diameter (mm)	Pass stricture?*
1 (M, 76y)	Hypopharynx	CRT; Rec: TLPE + RT	2 × transnasal balloon dilation	Neopharynx	15	Yes
2 (F, 66y)	1. Glottic larynx 2. Oro-/hypopharynx	1. RT 2. TLPE + CT	0	Neopharynx	15	No
3 (M, 87y)	Glottic larynx	RT; Rec: TLE	4 × bougienage under sedation	Neopharynx	11.3	No
4 (M, 73y)	Hypopharynx	1. RT; Rec: TLPE 2nd rec: no treatment	4 × bougienage under GA	Neopharynx	14	No
5 (M, 66y)	Glottic larynx	RT; Rec: TLE	12 × bougienage under GA	TE fistula	10	Yes
6 (M, 75y)	1. Glottic larynx 2. Hypopharynx	1. RT 2. TLPE	0	Proximal esophagus	15	Yes
7 (M, 71y)	Hypopharynx	TLPE	0	Neopharynx	15	Yes
8 (F, 71y)	Thyroid	Surgery + RT	0	Cricopharyngeal muscle	13.5	Yes
9 (M, 65y)	Glottic larynx	RT; Rec: TLE	1 × bougienage under GA	Neopharynx	15	Yes
10 (M, 65y)	Glottic larynx	RT; Rec: TLE + RT	0	Neopharynx	10	No
11 (M, 78y)	Hypopharynx	CRT	1 × bougienage under GA	Proximal esophagus	10	No
12 (M, 68y)	Supraglottic larynx	TLE	4 × bougienage under GA	Neopharynx	7.5	Yes

*M* male, *F* female, *y* years, *RT* radiotherapy, *CT* chemotherapy, *CRT* chemoradiotherapy, *TL(P)E* total laryng(opharyng)ectomy; *Rec* recurrence, *OB* office-based, *GA* general anesthesia

*Could the endoscope be passed through the stricture?

**Fig. 1:**
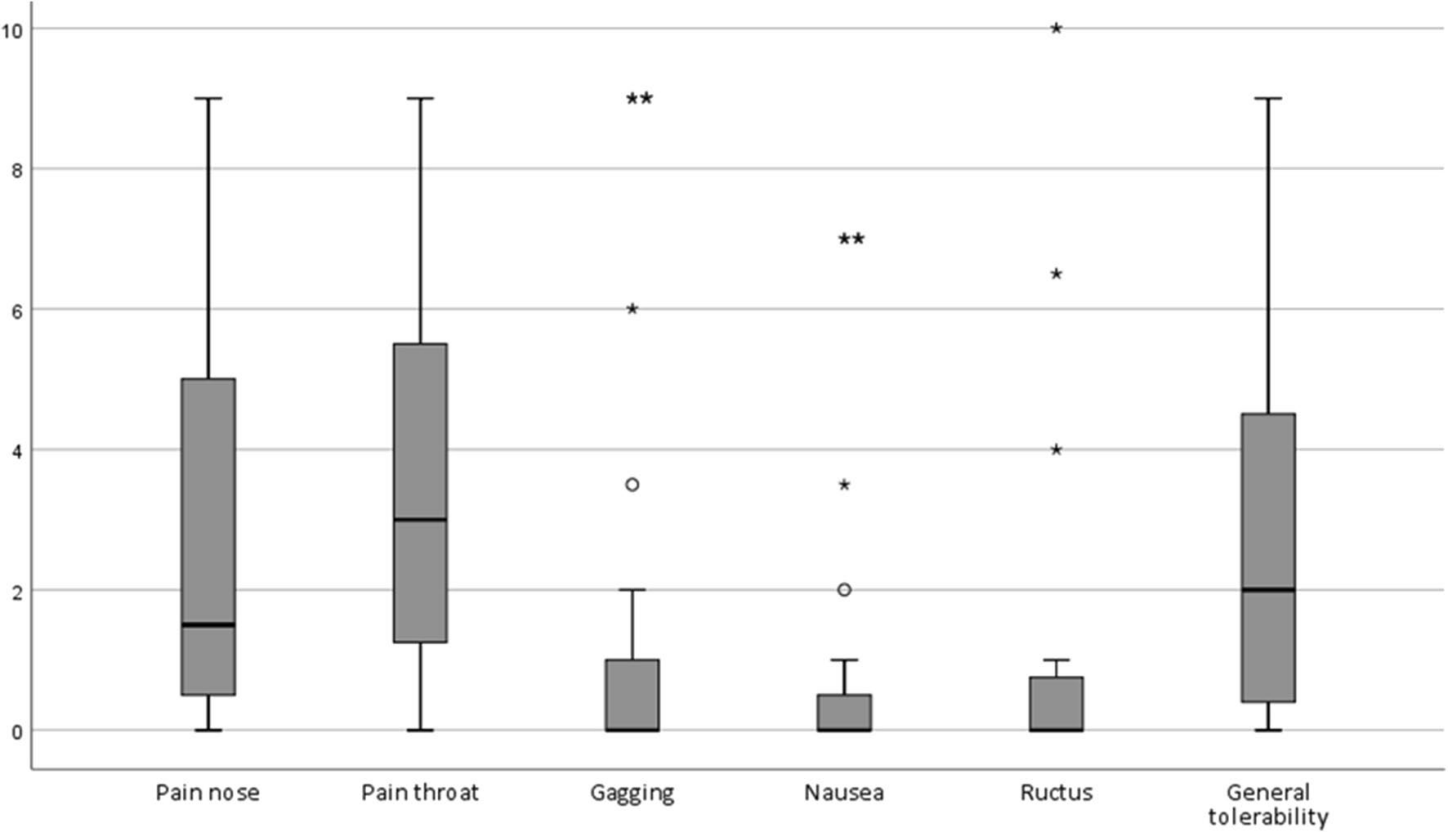
VAS scores on tolerance of the dilation procedure. Legend: Visual analogue scale (VAS): 0 = no complaints, 10 = unbearable complaints; ○ = outlier, value more between 1.5 – 3 times the Inter Quartile Range. * = extreme outlier, value more than 3 times the Inter Quartile Range

After each procedure, the patient rated the tolerability of the procedure using VAS scores (Fig. 1). Median VAS scores (0 = no complaints, 10 = unbearable complaints) on tolerability of the procedure were 3 or below in all topics. The general tolerability of the procedures was rated with a median VAS score of 2. The highest median VAS scores were for pain in the nose and throat. Figure 1 also shows that most patients had (almost) no complaints of gagging, nausea and ructus, but some outliers were observed.

The mean dysphagia score reduced from 2.1 (SD 1.2; range 1–5) pre-treatment to 1.7 (SD 1.4; range 0–5) 1 month post-treatment (p = 0.096) after a mean of 2.2 dilation procedures (SD 0.94; range 1–4). The mean maximum balloon diameter that was reached was 12.6 mm (SD 2.7; range 7.5–15).

**Fig. 2:**
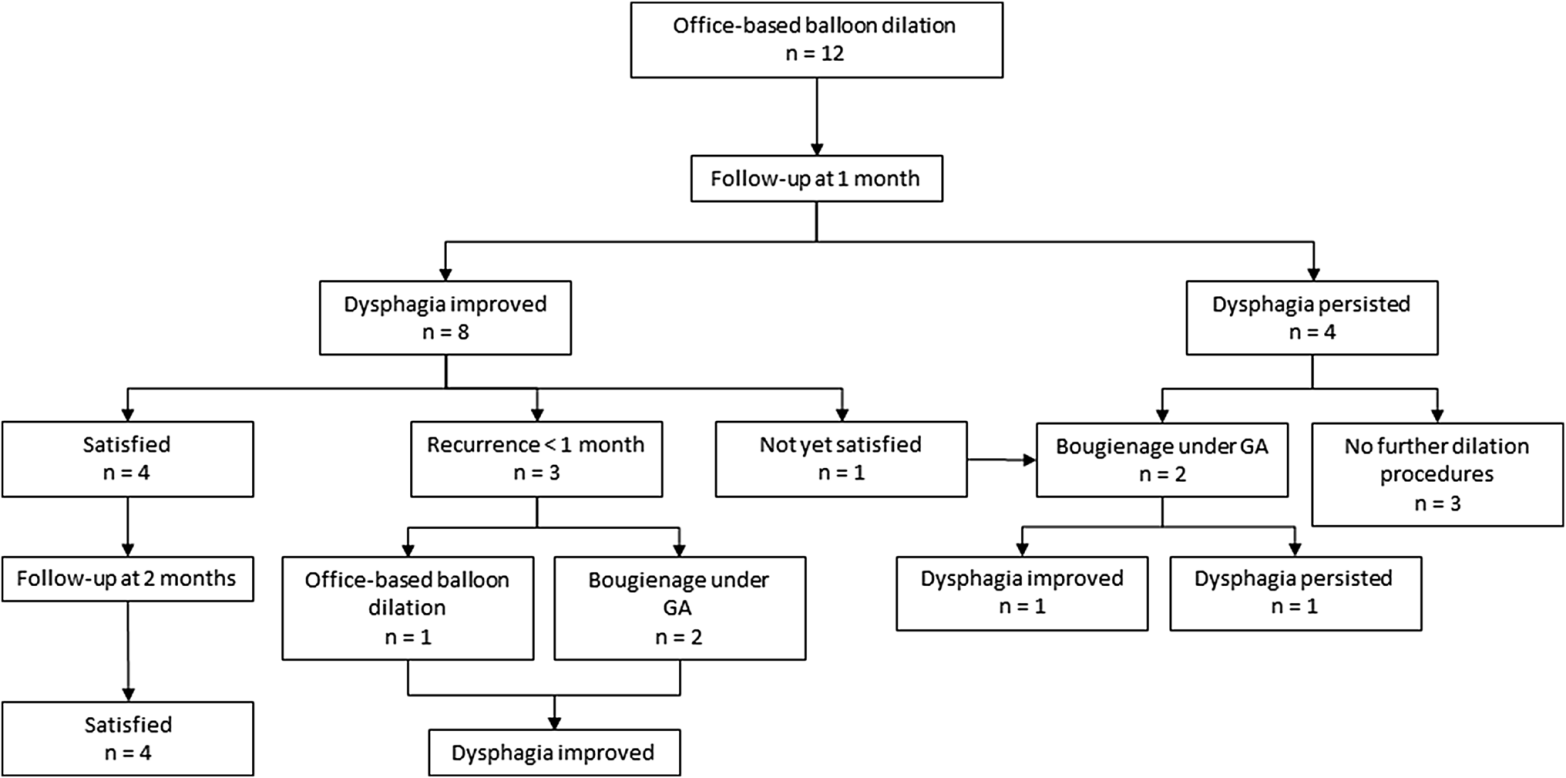
Flow chart of patient-reported treatment outcomes

The patient-reported outcomes of the transnasal balloon dilations are shown in Fig. 2. Eight patients (67%) reported that dysphagia improved after (a series of) transnasal dilation(s). Four of these patients previously underwent one or more dilations because of recurrent strictures. In the group of patients with unchanged dysphagia, 1 patient did not fully complete the dilation procedure (aborted after 1 inflation because of pain). This patient underwent bougienage under GA and hereafter dysphagia improved. Furthermore, 3 of these patients had a history of recurrent strictures and previous dilation(s) and in 2 patients dysphagia was not only caused by stricture formation, but also by the existence of a pseudovallecula and tongue motor dysfunction. None of the 4 successfully treated patients had a stricture that was too narrow for the endoscope to pass, whereas 5 of the 8 patients with recurrent or persistent dysphagia had an impassable stricture.

## Discussion

This prospective case series that analyzed 26 office-based transnasal balloon dilations performed in 12 head and neck cancer patients with neopharyngeal and proximal esophageal strictures, revealed high completion rate, low complication rate and good tolerability. The dilations improved dysphagia in 67% of the patients, although effects were transient in some patients.

So far, evidence for feasibility and safety of office-based transnasal balloon dilation was based on two retrospective studies [13, 14]. Completion rate of our study, which was the first known prospective study on this topic, was similar to the rate described by Rees et al. [13]. The low complication rate found in this study was in line with previously reported complication rates of 0% [13] and 10.6% [14]. Tolerability of the procedure has never been objectified in previous studies. By demonstrating low VAS scores on pain and discomfort, this study revealed that office-based transnasal balloon dilation was well-tolerated.

Stricture formation in patients previously treated for head and neck carcinoma is generally recurrent by nature [11]. Esophageal tissue is exposed to aggressive, in many cases multi-modality, oncological treatment which leads to severe circular fibrotic alterations. Although the mean dysphagia score improved after (a series of) dilation(s) in this study, the difference was not statistically significant. Also, the self-reported effectiveness was limited. The effectiveness of office-based transnasal balloon dilations has never been studied before, so no comparison with literature can be made. Agarwalla et al. described in their retrospective analysis on bougienage and balloon dilation of radiation-induced esophageal strictures that small (< 9 mm diameter) and radiation-induced strictures from prior head and neck carcinoma or metastatic disease were predictors of refractory strictures [17]. Additionally, patients with an anastomosis required more dilations compared to patients without anastomosis [17]. In this study, 42% of the patients had a stricture that was too narrow for the 5.1 mm diameter endoscope to pass, and 83% had a history of total laryng(opharyng)ectomy. In other words, our study population was at high risk for refractory strictures. Furthermore, 2 patients with persistent swallowing complaints had multilevel dysphagia. This resulted in unsatisfying effects of the dilation procedures and negatively influenced the outcomes of this study. Multilevel swallowing pathology can be a pitfall in the treatment of head and neck carcinoma patients with neopharyngeal or proximal esophageal strictures.

A strength of this study is its prospective study design, which reduces bias in reference to previous retrospective studies that were the only available evidence on office-based transnasal balloon dilation. Additionally, the population is homogeneous as it only consists of head and neck carcinoma patients with treatment related neopharyngeal and esophageal strictures. There are some limitations in this study. First, the study population was small. Limited number of patients were eligible for inclusion because the incidence of patients with strictures of the pharynx and proximal esophagus after treatment for head and neck carcinoma is relatively low. Furthermore, the follow-up was short because the main objective of this study was to assess feasibility and safety, not long term effectiveness. For a thorough analysis of effectiveness of transnasal balloon dilation, further research with larger cohorts is necessary.

This first prospective study on office-based transnasal balloon dilations in patients with a history of head and neck carcinoma demonstrated that this procedure is feasible, safe and well-tolerated. Transnasal balloon dilations can be used as an alternative to bougienage for patients with (recurrent) neopharyngeal or proximal esophageal strictures who have a contraindication for general anesthesia or prefer to undergo a procedure under topical anesthesia.
